# A comparison of central‐tendency and interconnectivity approaches to clustering multivariate data with irregular structure

**DOI:** 10.1002/ece3.9496

**Published:** 2022-11-18

**Authors:** Mark Tozer, David Keith

**Affiliations:** ^1^ NSW Department of Environment Parramatta New South Wales Australia; ^2^ Centre for Ecosystem Science, School of Biological, Earth and Environmental Science University of NSW Sydney New South Wales Australia

**Keywords:** Chameleon, classification, cluster metrics, cluster optimization, clustering, CLUTO, homogeneity, misplacement, vegetation databases

## Abstract

**Questions:**

Most clustering methods assume data are structured as discrete hyperspheroidal clusters to be evaluated by measures of central tendency. If vegetation data do not conform to this model, then vegetation data may be clustered incorrectly. What are the implications for cluster stability and evaluation if clusters are of irregular shape or density?

**Location:**

Southeast Australia.

**Methods:**

We define misplacement as the placement of a sample in a cluster other than (distinct from) its nearest neighbor and hypothesize that optimizing homogeneity incurs the cost of higher rates of misplacement. Chameleon is a graph‐theoretic algorithm that emphasizes interconnectivity and thus is sensitive to the shape and distribution of clusters. We contrasted its solutions with those of traditional nonhierarchical and hierarchical (agglomerative and divisive) approaches.

**Results:**

Chameleon‐derived solutions had lower rates of misplacement and only marginally higher heterogeneity than those of k‐means in the range of 15–60 clusters, but their metrics converged with larger numbers of clusters. Solutions derived by agglomerative clustering had the best metrics (and divisive clustering the worst) but both produced inferior high‐level solutions to those of Chameleon by merging distantly‐related clusters.

**Conclusions:**

Graph‐theoretic algorithms, such as Chameleon, have an advantage over traditional algorithms when data exhibit discontinuities and variable structure, typically producing more stable solutions (due to lower rates of misplacement) but scoring lower on traditional metrics of central tendency. Advantages are less obvious in the partitioning of data from continuous gradients; however, graph‐based partitioning protocols facilitate the hierarchical integration of solutions.

## Graphical abstract

Our results suggest that Chameleon may have an advantage over traditional algorithms at thematic scales at which data exhibit discontinuities and variable structure, potentially producing more stable solutions (due to lower rates of misplacement), but scoring lower on traditional metrics of central‐tendency. Chameleon's advantages are less obvious in the partitioning of continuous data, however its graph‐based partitioning protocol facilitates hierarchical integration of solutions.

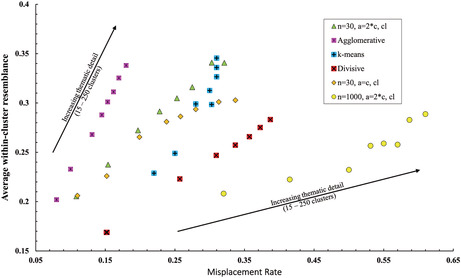

## 1 INTRODUCTION

Vegetation classification is the process of delimiting types of vegetation on the basis of their relative homogeneity and distinctness from other types (Van Der Maarel & Franklin, 2013). Classification facilitates not only the description of vegetation but also the study of its relationships with the environment and attendant interacting, co‐dependant organisms. Classification is thus the first step to the classification of ecosystems (*sensu* Tansley, 1935), and vegetation typologies have come to underpin a wide variety of conservation and natural resource management applications for terrestrial and coastal marine ecosystems including the selection of protected areas, ecosystem risk assessment and market‐based mechanisms such as biodiversity offsets (Bland et al., 2019). Despite a relatively short history, vegetation science has spawned a wide range of traditions (*sensu* Van Der Maarel & Franklin, 2013; Whittaker, 1975). Increasingly, however, vegetation classification centers on the clustering of quantitative plot samples (De Cáceres et al., 2015, 2018). When recorded with systematic procedures, plot samples have the advantage of allowing observations from different sources to be consolidated over time, while computer‐generated clustering solutions confer a degree of objectivity in the elucidation of patterns.

The utility of clustering in the development of vegetation classifications is beyond question, although it is complicated by three inter‐related problems. First, excepting simulated datasets, there is no agreed external point of reference with which clustering solutions can be compared. Instead, solutions based on field data must be evaluated on internal criteria (Aho et al., 2008), either geometric (e.g., cluster homogeneity) or nongeometric (e.g., species/cluster fidelity). Since these vary in the way they weigh particular characteristics of the solution, the best clustering solution may depend on its application. Second, the hyperspatial structure of vegetation data is generally unknown. The choice of both clustering algorithm and evaluation metrics therefore requires a user‐supplied model. This usually (but not invariably) assumes that clusters are spheroidal, or at least that it is appropriate to evaluate solutions based on within‐cluster homogeneity or other measures of central tendency (Aho et al., 2008; Lengyel et al., 2021). This is problematic because algorithms that seek to optimize central tendency can generate sub‐optimal solutions when applied to data with irregular structure, and internal metrics, which assume a spheroidal model may not be appropriate measures of cluster quality. Third, biases in both the geographic and environmental distribution of samples means that cluster metrics are often optimized for data that sample the range of floristic variation either unevenly or incompletely. That is, biases may induce irregularities in data structure even if assemblages in the field form a continuum. It is not surprising then, that clustering solutions are notoriously idiosyncratic and highly sensitive to data structure, transformations, choices of algorithm, and resemblance measures (Tichy et al., 2014). This limits their robustness to new data, and hence their stability for policy and management applications.

**FIGURE 1:**
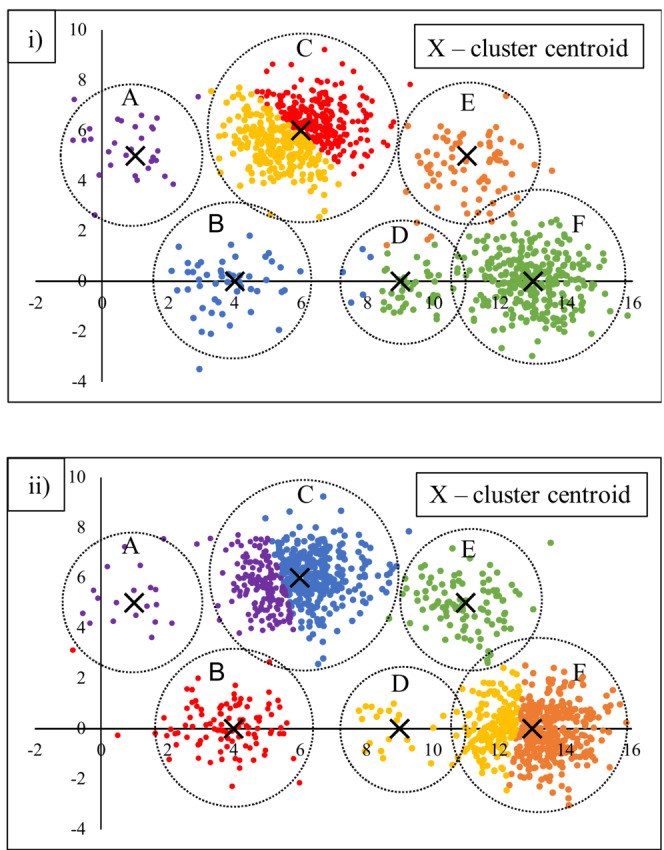
Sample clusters (A–F) Simulated data created by supplying cartesian coordinates for six centroids and generating random deviations from the centroids as bivariate standard normal errors with sample sizes (i) *n* = 30, 50, 500, 50, 70, 300), and (ii) *n* = 20, 100, 500, 20, 100, 500) with standard deviation = 1. The boundaries of each cluster are approximated by circles, colors indicate cluster membership as determined by k‐means operating on a matrix of Euclidean distances.

The potential limitations of assuming a spheroidal model to data of irregular structure are illustrated in Figure 1. The data are points on a cartesian plane, normally and randomly distributed around each of six predefined centroids. The k‐means algorithm fails to retrieve the underlying data structure; in (i) incorrectly splitting cluster C while merging clusters D and F; and in (ii) incorrectly splitting clusters C and F to partially merge with clusters A and D, respectively. The resulting solutions appear what Barton et al. (2019) termed “unnatural,” although they conceded the vagueness (*sensu* Regan et al., 2002) of circumscribing boundaries between clusters. Less subjectively, the solution is “incorrect,” for example, in Figure 1i in assigning samples that are co‐located in space in the region of centroid C to different groups, while drawing in remotely‐located samples from the region of centroid A. The implication is there is a high likelihood of alternative solutions arising as further data are added, or if the clustering algorithm is changed or supplied different parameters.

The problem illustrated in Figure 1 arises primarily from the insensitivity of the algorithm to variations in the density of points; however, a failure to recover “natural” or “correct” clusters of irregular shape has similarly been documented in a wide range of algorithms operating on assumptions of central tendency (Barton et al., 2019; Han et al., 2012; Karypis et al., 1999; Zhao & Karypis, 2005). The core principle underpinning algorithms which seek to retrieve clusters of irregular shape and/or density is sample interconnectivity. That is, cluster membership depends on interconnections among samples (based on pairwise similarity), rather than shared proximity to an artificial centroid or medoid. Schmidtlein et al. (2010), for example, noted two vegetation samples with no species in common could nevertheless share cluster membership provided they were connected in a chain of close neighbors. This implies clusters generated by an algorithm sensitive to irregular data structure are likely to be more heterogeneous than those derived with reference to a spheroidal model, particularly where discontinuities and variations in sample density exist.

Potential irregularities in the data structure are rarely accounted for in vegetation classification. Schmidtlein et al. (2010) documented a promising approach; however, our investigations of their ISOMAP algorithm suggested its “brute‐force” approach is too computationally demanding for a dataset comprising many thousands of samples (Schmidtlein et al., 2010). Lengyel and Botta‐Dukát (2019) introduced a generalization of the silhouette width index to allow for modifying its sensitivity to compactness versus connectedness. This modification avoids underestimating the efficacy of solutions that comprise more elongated and less compact clusters (provided their members are sufficiently interconnected); however, since the relevant parameter change is applied globally, it cannot easily accommodate data structures that include clusters with a range of shapes and densities.

Chameleon (Karypis et al., 1999, see methods for a detailed description) is one of several alternative algorithms designed to recover clusters of variable shape, which may, therefore, reproduce landscape‐scale relationships more faithfully than traditional clustering techniques (Han et al., 2012). Chameleon assesses both interconnectivity and closeness of objects as a basis for determining merging decisions, an approach that results in fewer “wrong” decisions than algorithms that consider only one or the other (Karypis et al., 1999). Focusing on interconnectivity allows the algorithm to adapt automatically to the characteristics of the clusters (density and hyperspatial distribution), rather than relying on a static model (e.g., discrete spherical clusters or degrees of compactness). Therefore, provided they are strongly interconnected, samples spanning a compositional continuum can be retrieved as a single cluster even if the distribution of samples along the continuum is uneven, because Chameleon is relatively insensitive to variations in hyperspatial density (Han et al., 2012).

We suggest that a failure to take account of the underlying structure of vegetation data is likely to be one factor contributing to idiosyncrasies among clustering solutions; however, the effect is likely to be dependent on the expression and nature of discontinuities in the data structure. We postulate that accounting for the data structure is more likely to be important at broad levels of classification (lower numbers of clusters, as represented by the points in Figure 1 collectively) because discontinuities are likely to arise both naturally (e.g., between regions that share few species), due to variable data coverage (De Cáceres et al., 2018, Gellie et al., 2018) or because environmental gradients are discontinuous in geographic space (Austin, 2013). Conversely, there may be no disadvantage in assuming a spheroidal model where clustering essentially amounts to partitioning a continuum (i.e., partitioning the individual clusters in Figure 1).

In this paper, we investigate two hypotheses: (i) that an algorithm sensitive to hyperspatial irregularities in the density and arrangement of samples will produce clusters that are likely to be more “correct” (in the sense that samples are co‐located with their close neighbors) but at the cost of poorer internal metrics relative to algorithms that seek to optimize around central tendency; and (ii) differences between the respective algorithms will decline with the increasing number of clusters. To test these hypotheses, we used a large regional dataset of 7541 plot samples to compare the performance of traditional clustering algorithms (k‐means, hierarchical agglomerative, and divisive) with the Chameleon algorithm. For this evaluation, we used both internal metrics (homogeneity, indicator species) and the concept of “correctness,” which we apply as the misplacement rate: the proportion of samples that do not cluster with their nearest neighbor. Since we could find no examples of the use of Chameleon in the clustering of vegetation data, we explore the effects of different parameter settings and compare Chameleon's clusters (15‐cluster solution) to the units of a reference classification.

## 2 METHODS

### 2.1 The Chameleon algorithm

**FIGURE 2:**
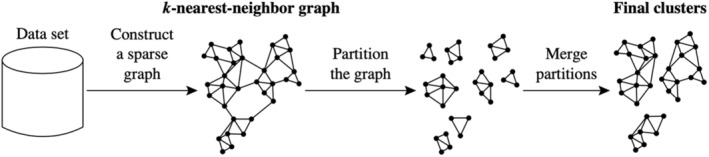
Graphical representation of Chameleon's two‐phase algorithm (reproduced from Karypis et al., 1999)

Chameleon models the feature space as a k‐nearest neighbor graph (sparse graph) with samples forming vertices connected by links that are proportional to pairwise similarity between samples (Figure 2). The user specifies the number of links between samples (neighborhood range), and then in the first phase, links are progressively dissolved (in order of increasing similarity) until a user‐specified number of sub‐partitions has formed. In this partitioning phase, the algorithm seeks to minimize the summed length of all dissolved links, hence minimizing the affinity between samples in different sub‐partitions (Karypis et al., 1999). Sub‐partitions are then (optionally) merged using a hierarchical agglomerative clustering algorithm to resolve the number of groups required for the solution. An advantage of this approach is that it encapsulates the concept of environmental/compositional continua by weighting cluster interconnectivity over homogeneity. That is, samples that are distantly related may still share a cluster if they are linked by strongly interconnected neighbors. One of the key features of the Chameleon algorithm is that the structure of intersample relationships is preserved through the partitioning phase because the co‐membership of sub‐partitions is dependent on pairwise intersample connectivity. By contrast, traditional algorithms merge or split samples progressively and the outcome at each step depends on comparing samples with intermediate clusters, the compositional characteristics of which are artificial and reflect the range of the samples merged (Han et al., 2012).

### 2.2 Study area

The study area encompassed the South East Highlands and Australian Alps Bioregions of the state of NSW, Australia (Thackway & Creswell, 1995), an area of 96,089 km^2^ encompassing mountains and plateaus of the Great Dividing Range. Average annual precipitation ranges from 460 to 2,344 mm and mean annual temperatures are 3 – 16^o^ C. The area is underlain by a complex series of heavily folded metamorphosed sedimentary rocks deposited from the Ordovician to Devonian periods and interspersed with numerous granite intrusions and, to a much lesser extent, basalts deposited in the Paleogene.

Primary factors influencing the distribution of vegetation formations in our study area include temperature, rainfall, topography, soils, and drainage (Beadle, 1981; Costin, 1954; Jenny, 1983; Keith, 2004). Alpine assemblages are restricted to elevations more than 1830 m above sea level where winter temperature minima fall below the physiological tolerance of trees (Keith, 2004). Tree cover progressively increases with decreasing elevations as the severity of winter conditions declines. Sub‐alpine grassy woodlands occupy the sub‐alpine tracts, characteristically with short, gnarled trees and a large compliment of cold‐tolerant species also found in the alpine zone. On the southwest flank of the Great Divide, sub‐alpine woodlands grade into tall wet sclerophyll forests, sustained by high orographic rainfall originating in south‐westerly air flows. To the east, depending on soil lithology texture and fertility, sub‐alpine woodlands grade into either Dry Sclerophyll Forest or Grassy Woodlands as annual rainfall declines in the shadow of the Divide. Grasslands replace Grassy Woodlands in frost hollows, the heaviest‐texture soils, and the most moisture‐limited sites (Costin, 1954). Further east of the tablelands, grasslands, and grassy woodlands are replaced by mosaics of wet and dry sclerophyll forests on the escarpment ranges as rainfall increases with increasing elevation and exposure to oceanic weather systems (Keith, 2004). Wetlands occur throughout the bioregions in areas of impeded drainage, while heathlands are among the local expressions of edaphic and topographic variation.

### 2.3 Compilation of floristic data

We sourced a total of 7541 floristic plot samples from a database compiled and administered by the Department of Planning, Industry and Environment (DPIE, 2019). These comprised all survey data collected in (or within 25 km of) the South East Highlands and Australian Alps Bioregions, which met the following criteria: (i) the sample location was recorded with an accuracy of 100 m; (ii) the sample area was 0.04 ha; and (iii) all vascular plant species were recorded. Individual species records were reviewed and modified to resolve inconsistencies in taxonomy (see Methods in Tozer et al., 2010). Taxa identified only at the generic level were removed along with records of naturalized species. Cover‐abundance scores were transformed to presence‐absence to eliminate the possible effects of bias in cover‐abundance estimates by different observers. This transformation was considered an appropriate strategy to achieve a balance between information loss and maximizing the pool of available data in circumstances where the dataset is both large and likely to be heterogeneous (Goodall, 1978).

### 2.4 Chameleon performance evaluation

We performed all Chameleon analyses on pairwise Bray‐Curtis compositional similarities (also known as Sörensen(‐Dice) index for presence‐absence data) between samples (Clarke, 1993) using the scluster function in CLUTO software version 2.1.2 (Karypis, 2003). First, since we found little information in the literature to guide parameter‐setting, we assessed solutions of 15 clusters over a range of neighborhood sizes (15–1000 neighbors), degrees of sub‐partitioning (up to 500 sub‐partitions or agglomerative phase omitted), and linkage functions (single or complete) (Table 1). We carried out our initial trials using the single‐link criterion function in the agglomerative phase, as recommended for nonspherical clusters (Karypis et al., 1999). For each solution, we calculated the average pairwise within‐cluster similarity (homogeneity) and the proportion of samples located in clusters other than that of their nearest neighbor (misplacement rate). Specifying more than 30 sub‐partitions caused extensive chaining (*sensu* Peet & Roberts, 2013). We repeated the relevant trials using an option forcing Chameleon to prioritize large clusters over small ones in the partitioning phase as recommended to counter a tendency to chaining in a solution (Karypis et al., 1999). On the basis of the preliminary results, we undertook subsequent analyses using the complete linkage function and assessed performance over a range of cluster numbers (15–250 clusters) and degrees of sub‐partitioning (30–500 sub‐partitions) with neighborhood size fixed at either 30 or 1000 (Table 1).

**TABLE 1:** Summary of analytical trials undertaken, their purpose, and results. Colors mentioned in column 1 match indicative results plotted in Figures 4, 5, 6, 7

Algorithm	Number of clusters	Neighborhood size (Chameleon only)	Number of sub‐partitions (Chameleon only)	link	purpose	Examples results in figures	result
Chameleon (white)	15	15 ‐ 1000	30, 60, 90, 150	single	Assess performance under different combinations of neighborhood size and number of sub‐partitions	3, 5	chaining increased with increasing number of sub‐partitions over 30, misplacement rate increased with neighborhood size and no clear patterns in within‐cluster homogeneity with either neighborhood size or number of sub‐partitions
Chameleon (Brown)	15	15 ‐ 1000	agglomerative phase omitted	NA	Assess performance under different neighborhood sizes with no agglomerative phase	5	misplacement rate decreased strongly, within‐cluster homogeneity decreased weakly with increasing neighborhood size
Chameleon (Pink)	15	30	30, 60, 120, 180, 240, 300, 400, 500	complete	Assess effect of increasing number of sub‐partitions on a 15‐cluster solution with neighborhood size of 30 samples	5, 7	misplacement rate decreased weakly, within‐cluster homogeneity decreased strongly with increasing neighborhood size
Chameleon (Orange)	15, 30, 60, 90, 120, 150 200, 250	30	agglomerative phase omitted	NA	Assess performance on cluster solutions with different numbers of clusters, neighbor size fixed, agglomerative phase omitted	6, 7	misplacement rate and within‐cluster homogeneity increased with increasing classification detail, cluster solutions relatively even in size
Chameleon (Green)	15, 30, 60, 90, 120, 150 200, 250	30	15, 30, 60, 120, 180, 240, 300, 400, 500	complete	Assess performance on cluster with different numbers of clusters, neighbor size fixed, number of sub‐partitions proportional to number of final clusters	6, 7	misplacement rate and within‐cluster homogeneity increased with increasing classification detail, cluster solutions relatively even in size
Chameleon (Yellow)	15, 30, 60, 90, 120, 150 200, 250	1000	30, 60, 120, 180, 240, 300, 400, 500	complete	Assess performance on cluster with different numbers of clusters, neighbor size fixed, number of sub‐partitions proportional to number of final clusters	7	misplacement rate and within‐cluster homogeneity increased with increasing classification detail, cluster solutions relatively even in size
k‐means (Blue)	15, 30, 60, 90, 120, 150 200, 250	NA	NA	NA	Assess performance of k‐means algorithm over cluster solutions with different numbers of clusters	5, 6, 7	misplacement rate and within‐cluster homogeneity increased with increasing classification detail, cluster solutions relatively even in size
flexible unweighted pair‐group averaging with arithmetic mean (Belbin et al., 1992) (Purple)	15, 30, 60, 90, 120, 150 200, 250	NA	NA	average	Assess performance of agglomerative algorithm over cluster solutions with different numbers of clusters	5, 6, 7	misplacement rate and within‐cluster homogeneity increased with increasing classification detail, cluster solutions relatively uneven in size
polythetic‐division (Belbin et al., 1984; MacNaughton‐Smith et al., 1964) (Red)	15, 30, 60, 90, 120, 150 200, 250	NA	NA	average	Assess performance of divisive algorithm over cluster solutions with different numbers of clusters	5, 6, 7	misplacement rate and within‐cluster homogeneity increased with increasing classification detail, cluster solutions relatively uneven in size

### 2.5 Comparison of algorithms

A very wide range of algorithms has been applied to the classification of vegetation data, with variable performance as measured by internal and external metrics such as cluster homogeneity, silhouette width, species fidelity, etc. (e.g., Aho et al., 2008; Lengyel et al., 2021). These algorithms vary both in complexity and in the extent they have been applied, but since they are generally applied with the expectation of retrieving compact clusters, we sought to compare our alternative approach with traditional, widely‐understood approaches (Kent, 2011). We compared Chameleon cluster member sets with those derived from: (i) k‐means clustering (Belbin, 1987; MacQueen, 1967); (ii) unweighted pair‐group method with arithmetic means (Belbin et al., 1992); and (iii) polythetic division (MacNaughton‐Smith et al., 1964; Belbin et al., 1984), all implemented using the PATN package (Belbin, 1987). We used each algorithm to compute solutions ranging from 15 to 250 clusters (Table 1.) and characterized solutions in terms of homogeneity and misplacement rate (as described above), the number of species occurring at higher frequencies within each cluster than in the dataset as a whole (cumulative hypergeometric probability >0.999), and the number of species with standardized phi > 0.35 (Tichý & Chytrý, 2006).

### 2.6 Comparing clustering solutions with a reference classification

We assessed the extent to which clustering solutions (15 classes) produced by each algorithm retrieved species sets characterizing the units of an established subcontinental‐scale vegetation classification that covers 800,000 km^2^ in southeastern Australia (Keith, 2004), including the study area (c. 11% of total area). The reference classification was developed from the top down based on an extensive review of vegetation studies, field reconnaissance, and qualitative synthesis of vegetation maps available at the time (Keith, 2004). Its highest level of classification (vegetation formation) is based on structural/physiognomic features. Formations are subdivided into vegetation classes based on geographically distinct expressions of structural and compositional features. Fifteen of 99 vegetation classes recognized in the reference classification are mapped within the study area and are described with lists of indicative species (Keith, 2004). For each clustering solution, we identified the species diagnostic of each cluster as those with a frequency of occurrence statistically higher within the cluster samples than across the dataset as a whole (cumulative hypergeometric probability > 0.999). We compared these with the species identified as diagnostic of the reference classes, compiling a confusion matrix with the units of the respective classifications as rows and columns and cell values calculated as the proportion of reference class species that were identified as diagnostic of each cluster class.

## 3 RESULTS

**FIGURE 3:**
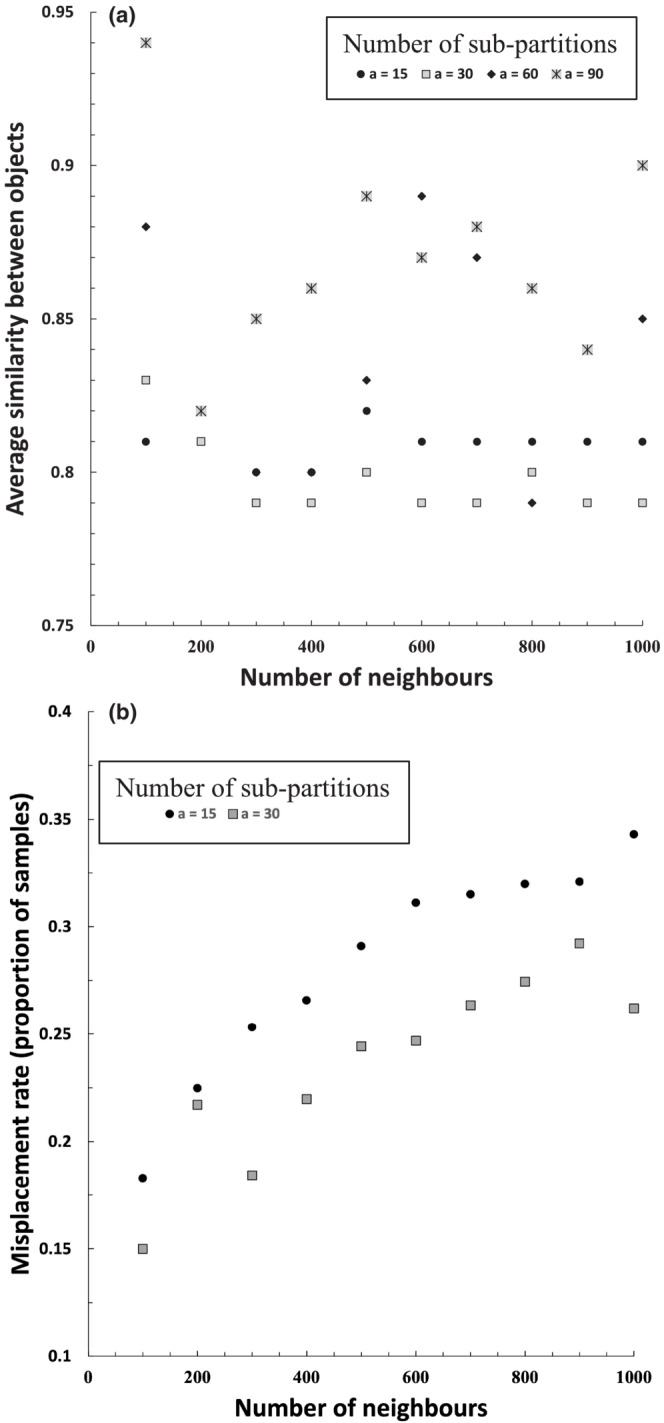
Misplacement rate (a) and average similarity among objects within clusters (b) as a function of neighborhood size. Results for solutions obtained with more than 30 sub‐partitions are not shown in (a) because samples were frequently concentrated in a single cluster (chaining). Trials incorporating an agglomeration phase (a > 15) were performed using a single‐linkage function.

**FIGURE 4:**
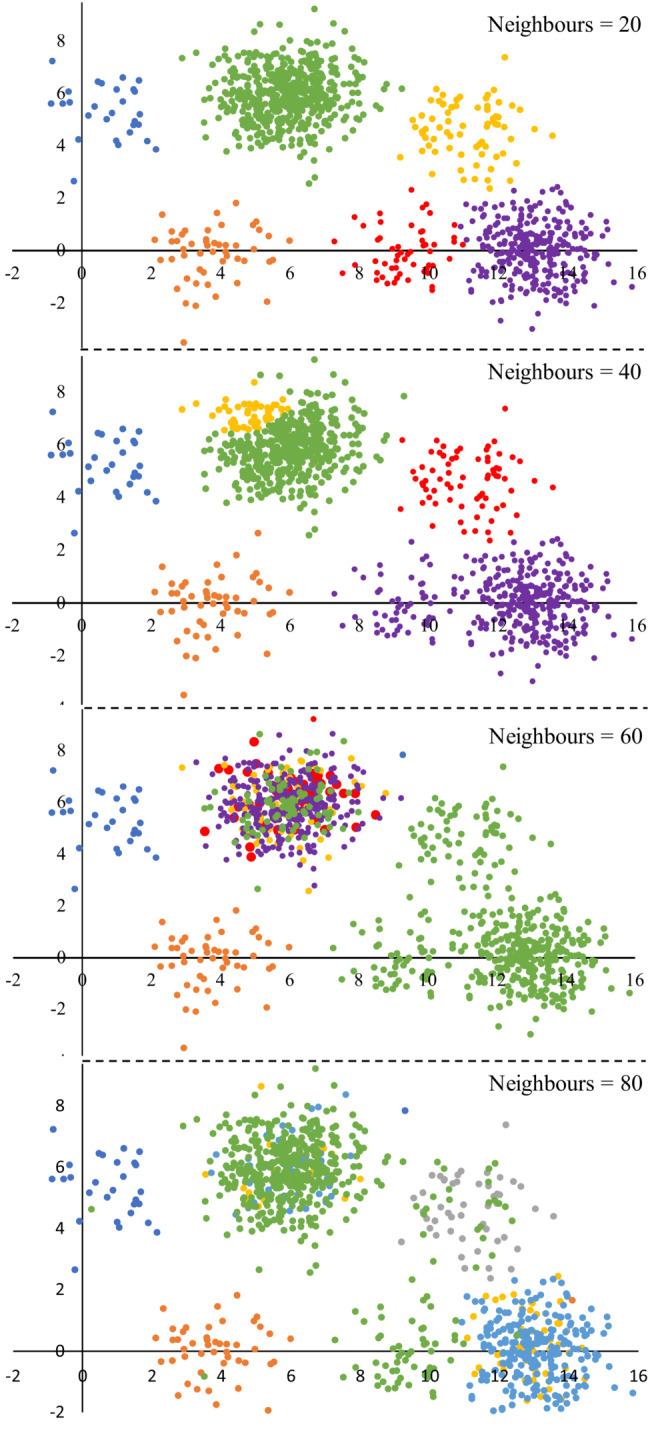
Clustering of simulated data (Figure 1) by Chameleon illustrating increasing rates of misplacement with increasing neighborhood size using the single‐linkage function. Samples with the same color were placed in the same cluster.

A summary of the analytical trials performed and a brief synopsis of the results are contained in Table 1. A detailed description of the results follows:

Trends in the misplacement rate and average within‐cluster homogeneity in Chameleon cluster solutions generated using the single‐link functions are summarized in Figure 3. The misplacement rate rose with increasing neighborhood size (Figure 3a). This result may reflect aberrations caused by forcing members of small clusters to forge links with samples in other clusters as illustrated by Chameleon's attribution of the simulated data we presented in Figure 1 given neighborhoods of different sizes (Figure 4). Solutions derived by agglomeration from 30 sub‐partitions had consistently lower rates of misplacement, but solutions with more sub‐partitions became increasingly uneven (chaining) and misplacement rates became meaningless because a high proportion of samples were concentrated in few clusters. The problem of chaining was not corrected by directing the algorithm to prioritize large clusters (over small) in the partitioning phase; however, more even clusters were produced when the complete link function was employed in the agglomerative phase of the algorithm and subsequent analyses were performed using this option, as described in the next section. There was no clear trend in within‐cluster homogeneity with increasing neighborhood size when the agglomeration phase was omitted (Figure 3b). Solutions derived by agglomeration from 30 sub‐partitions had the lowest homogeneity with a neighborhood size of 100. Beyond 30 sub‐partitions the data showed no clear trend and varied erratically depending on the unevenness of the solutions.

**FIGURE 5:**
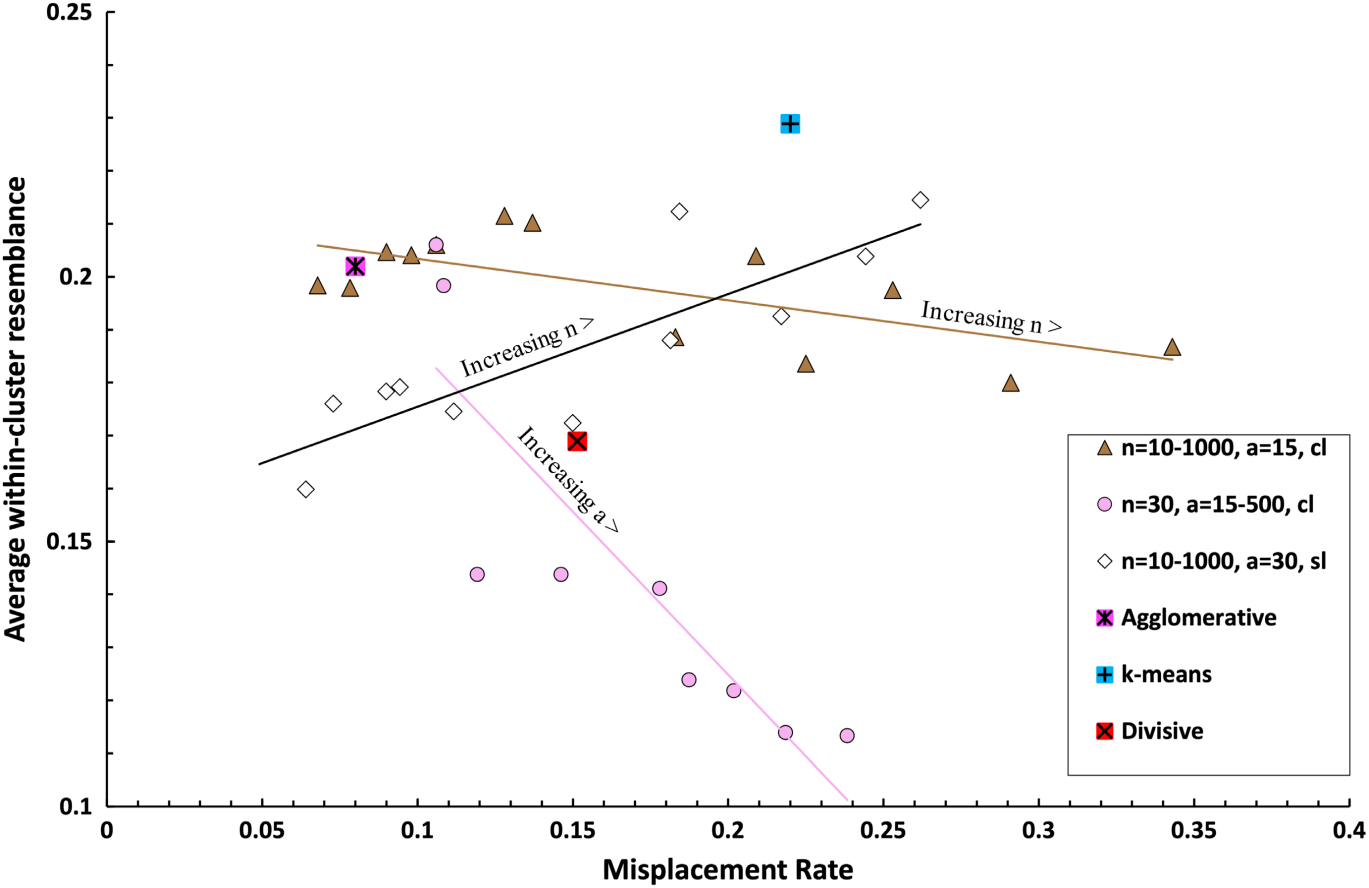
Trends in misplacement rate and within‐cluster homogeneity with increasing neighborhood size or increasing number of sub‐partitions in the agglomeration phase. The effect of increasing sub‐partitions using the single‐linkage function is not shown due to chaining as described above). Trendlines are least‐squares linear regressions. Data describing the respective 15‐cluster solutions derived by k‐means, agglomerative, and divisive algorithms are plotted for comparison (see Figure 6) (cl, complete linkage; sl, single‐linkage).

Clusters of 15 solutions generated using the complete link function exhibited higher rates of misplacement and lower within‐cluster homogeneity when either neighborhood size (*n*) or the number of sub‐partitions (*a*) in the agglomerative phase were increased, although increasing *n* disproportionately affected the misplacement rate while increasing *a* disproportionately affected cluster homogeneity (Figure 5).

**FIGURE 6:**
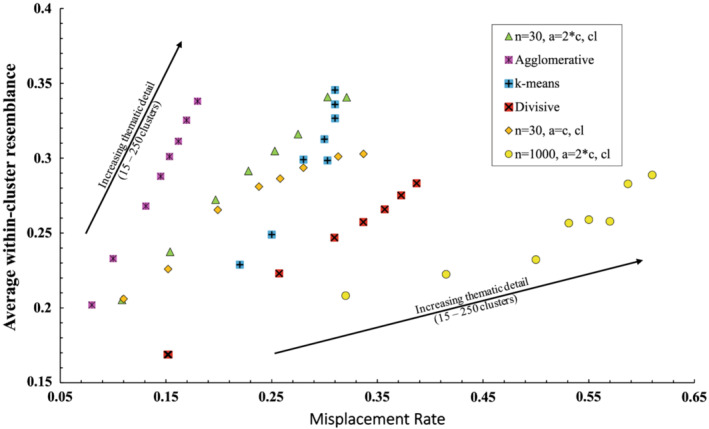
Trends in misplacement rate and within‐cluster homogeneity with increasing classification detail (15–250 clusters) (cl, complete linkage)

**FIGURE 7:**
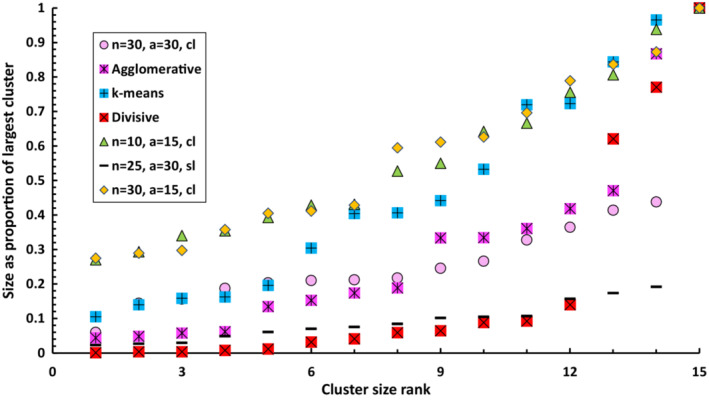
Cluster sizes ranked in order of increasing size and plotted as a proportion of the number of samples in the largest cluster

Both the rate of misplacement and within‐cluster homogeneity increased with increasing numbers of clusters (Figure 6). Chameleon solutions derived using small neighbor sizes and either: modest numbers of sub‐partitions (twice the number of classes in the solution); or with the agglomeration phase omitted, were better (lower rates of misplacement and higher homogeneity) than those derived with the divisive algorithm but worse than those derived with the agglomerative algorithm (Figure 6). However, 15‐ class solutions derived by Chameleon were more even (i.e., the clusters had similar numbers of members) than those produced by either the agglomerative or divisive algorithms (Figure 7). Chameleon solutions were better than those of k‐means in broad classifications (15–60 classes) but equivalent at finer classifications (90–250 classes). Chameleon produced more even 15‐class solutions than k‐ means (Figure 7).

Clusters derived by Chameleon solutions were generally characterized by fewer diagnostic species than those derived using the traditional algorithms (Table 2); however, species diagnostic of Chameleon clusters corresponded more with those characterizing units of a reference classification for our study area than those diagnostic of clusters derived by agglomerative or divisive algorithms, both in the range of units represented and with less overlap between unrelated units (Tables 3, 4, 5). Clusters derived by k‐means retrieved units of the reference classification with efficiency similar to Chameleon (Table 6).

**TABLE 2:** Total number of diagnostic species across all classes as determined by frequency (statistically higher than background frequency) or standardized phi (Tichý & Chytrý, 2006)

Algorithm	Total number of species with class hypergeometric probability > 0.999	Total number of species with class hypergeometric probability > 0.999 AND class frequency >= 0.3 (median frequency)	Total number of species with PHI >= 0.35	Total number of species with PHI >= 0.35 AND class frequency >= 0.3 (median frequency)
k‐means	3615	303 (0.42)	203	121 (0.44)
Agglomerative	3478	282 (0.42)	227	125 (0.43)
Divisive	3118	278 (0.45)	222	144 (0.44)
Chameleon (*n* = 10, a = 15)	3569	252 (0.42)	123	66 (0.41)
Chameleon (*n* = 40, a = 15)	3572	268 (0.41)	123	66 (0.42)
Chameleon (*n* = 100, a = 15)	3416	269 (0.44)	55	31 (31)
Chameleon (*n* = 1000, a = 15)	3416	269 (0.44)	60	34 (34)
Chameleon (*n* = 1000, a = 60)	4646	549 (0.44)	173	115 (115)

**TABLE 3:** Proportion of characteristic species for each reference class (rows) shared with clusters from the Chameleon algorithm (15 clusters based on neighborhood range of 1000 samples agglomerated from 30 sub‐partitions)

Cluster (15)	6	7	9	0	4	5	8	14	3	15	1	12	2	10	11	13
Alpine Herbfields	0.88	0.00	0.00	0.12	0.19	0.19	0.19	0.00	0.00	0.00	0.00	0.19	0.08	0.00	0.00	0.04
Alpine Bogs and Fens	1.00	0.00	0.00	0.04	0.04	0.07	0.04	0.11	0.04	0.00	0.00	0.21	0.04	0.04	0.00	0.04
Alpine Heaths	0.93	0.00	0.07	0.07	0.07	0.00	0.00	0.04	0.04	0.00	0.00	0.37	0.04	0.00	0.04	0.00
Alpine Fjaeldmarks	1.00	0.00	0.00	0.00	0.00	0.06	0.00	0.06	0.06	0.00	0.00	0.06	0.00	0.00	0.00	0.00
Southern Tablelands DSF	0.03	0.80	0.14	0.40	0.43	0.31	0.09	0.06	0.06	0.09	0.23	0.00	0.26	0.06	0.31	0.09
Southern Escarpment WSF	0.00	0.03	0.88	0.38	0.16	0.00	0.00	0.00	0.00	0.16	0.00	0.03	0.16	0.31	0.06	0.00
Montane WSF	0.11	0.09	0.49	0.66	0.20	0.06	0.06	0.06	0.06	0.14	0.06	0.14	0.11	0.03	0.03	0.00
Southern Tableland WSF	0.02	0.17	0.41	0.68	0.66	0.27	0.15	0.00	0.02	0.12	0.05	0.07	0.44	0.20	0.39	0.15
Sub‐alpine woodlands	0.19	0.24	0.19	0.62	0.57	0.22	0.14	0.03	0.05	0.08	0.08	0.11	0.30	0.05	0.22	0.05
Tableland Clay GW	0.08	0.05	0.14	0.41	0.65	0.46	0.46	0.00	0.03	0.03	0.00	0.14	0.38	0.14	0.41	0.14
Southern Tablelands GW	0.00	0.26	0.14	0.42	0.79	0.74	0.49	0.02	0.00	0.05	0.02	0.02	0.47	0.14	0.51	0.26
Temperate Montane grasslands	0.07	0.04	0.00	0.19	0.48	0.70	0.85	0.00	0.00	0.00	0.00	0.22	0.44	0.00	0.22	0.11
Southern Montane Heaths	0.03	0.24	0.07	0.10	0.14	0.07	0.03	0.59	0.38	0.21	0.28	0.07	0.03	0.07	0.00	0.00
Sydney Montane Heaths	0.00	0.08	0.00	0.04	0.04	0.04	0.00	0.42	0.92	0.50	0.25	0.00	0.00	0.04	0.04	0.00
Sydney Montane DSF	0.00	0.20	0.10	0.07	0.10	0.00	0.00	0.13	0.63	0.80	0.43	0.00	0.10	0.00	0.07	0.00
South East DSF	0.04	0.37	0.33	0.11	0.11	0.02	0.00	0.20	0.43	0.65	0.50	0.00	0.17	0.15	0.09	0.00
Montane Bogs and Fens	0.49	0.04	0.23	0.05	0.15	0.04	0.09	0.32	0.21	0.06	0.02	0.72	0.04	0.00	0.02	0.06
Montane Lakes	0.23	0.00	0.00	0.05	0.09	0.05	0.09	0.00	0.00	0.00	0.00	1.00	0.00	0.00	0.00	0.00
Central Gorge DSF	0.00	0.20	0.04	0.15	0.14	0.16	0.11	0.20	0.02	0.18	0.23	0.00	0.75	0.68	0.27	0.14

*Note*: Dark gray indicates proportions > 0.7 and pale gray proportions > 0.5. Cells with the same shading in column one comprise members of the same formation.

Abbreviations: DSF, Dry Sclerophyll Forests; GW, Grassy Woodlands; WSF, Wet Sclerophyll Forests.

**TABLE 4:** Proportion of species characteristic of each structural/physiognomic class that are diagnostic of units of a 15‐cluster solution derived using polythetic division (DSF ‐ Dry Sclerophyll Forests; WSF ‐ Wet Sclerophyll Forests; GW ‐ Grassy Woodlands)

Cluster	2	1	11	8	7	5	9	3	4	6	10	12	13	14	15
Alpine Herbfields	0.73	0.00	0.00	0.15	0.23	0.00	0.00	0.35	0.27	0.00	0.00	0.04	0.00	0.00	0.00
Alpine Bogs and Fens	0.57	0.00	0.00	0.07	0.04	0.07	0.00	0.79	0.25	0.00	0.00	0.00	0.00	0.00	0.00
Alpine Heaths	0.89	0.00	0.07	0.26	0.00	0.04	0.00	0.26	0.11	0.04	0.04	0.07	0.04	0.07	0.07
Alpine Fjaeldmarks	0.88	0.00	0.00	0.00	0.06	0.06	0.00	0.31	0.06	0.00	0.00	0.06	0.00	0.00	0.00
Southern Tablelands DSF	0.00	0.80	0.14	0.43	0.26	0.00	0.03	0.00	0.00	0.14	0.03	0.03	0.03	0.06	0.06
Southern Escarpment WSF	0.03	0.06	0.91	0.59	0.00	0.00	0.44	0.00	0.03	0.41	0.03	0.13	0.22	0.13	0.22
Montane WSF	0.14	0.14	0.40	0.83	0.06	0.03	0.14	0.00	0.09	0.20	0.03	0.03	0.11	0.09	0.11
Southern Tableland WSF	0.02	0.20	0.34	0.83	0.44	0.00	0.24	0.00	0.15	0.54	0.07	0.17	0.29	0.27	0.32
Sub‐alpine woodlands	0.22	0.24	0.14	0.78	0.24	0.00	0.05	0.00	0.08	0.27	0.05	0.08	0.11	0.16	0.16
Tableland Clay GW	0.08	0.08	0.14	0.57	0.59	0.03	0.08	0.03	0.14	0.27	0.00	0.05	0.16	0.16	0.11
Southern Tablelands GW	0.00	0.21	0.12	0.49	0.79	0.00	0.12	0.00	0.05	0.28	0.05	0.09	0.21	0.21	0.16
Temperate Montane grasslands	0.04	0.04	0.00	0.30	0.89	0.00	0.00	0.04	0.19	0.11	0.00	0.00	0.00	0.07	0.00
Southern Montane Heaths	0.00	0.76	0.00	0.14	0.07	0.21	0.07	0.00	0.03	0.03	0.00	0.00	0.03	0.00	0.00
Sydney Montane Heaths	0.00	0.67	0.04	0.04	0.04	0.92	0.00	0.04	0.00	0.04	0.04	0.04	0.04	0.00	0.00
Sydney Montane DSF	0.00	0.97	0.07	0.10	0.03	0.33	0.03	0.00	0.00	0.10	0.00	0.03	0.07	0.00	0.00
South East DSF	0.02	0.85	0.28	0.13	0.07	0.26	0.15	0.00	0.00	0.24	0.02	0.09	0.20	0.17	0.11
Montane Bogs and Fens	0.13	0.04	0.00	0.32	0.13	0.19	0.00	0.64	0.74	0.06	0.00	0.04	0.00	0.02	0.02
Montane Lakes	0.05	0.00	0.00	0.05	0.14	0.00	0.00	0.14	1.00	0.00	0.00	0.05	0.00	0.05	0.00
Central Gorge DSF	0.00	0.27	0.07	0.07	0.59	0.00	0.50	0.00	0.00	0.43	0.05	0.09	0.39	0.27	0.11

*Note*: Dark gray indicates proportions > 0.7 and pale gray proportions > 0.5. Cells with the same shading in column one comprise members of the same formation.

Abbreviations: DSF, Dry Sclerophyll Forests; GW, Grassy Woodlands; WSF, Wet Sclerophyll Forests.

**TABLE 5:** Proportion of species characteristic of each structural/physiognomic class that are diagnostic of units of a 15‐cluster solution derived using pairwise unweighted group averaging) (DSF ‐ Dry Sclerophyll Forests; WSF ‐ Wet Sclerophyll Forests; GW ‐ Grassy Woodlands)

Cluster	14	13	15	7	4	5	11	1	2	8	10	6	3	9	12
Alpine Herbfields	0.58	0.46	0.46	0.12	0.00	0.15	0.00	0.19	0.15	0.04	0.00	0.00	0.04	0.04	0.00
Alpine Bogs and Fens	0.50	0.79	0.43	0.07	0.00	0.18	0.00	0.07	0.00	0.00	0.11	0.00	0.04	0.04	0.00
Alpine Heaths	0.85	0.30	0.56	0.07	0.07	0.41	0.07	0.07	0.00	0.04	0.07	0.00	0.00	0.00	0.04
Alpine Fjaeldmarks	0.25	0.31	0.88	0.00	0.00	0.00	0.00	0.00	0.06	0.00	0.06	0.00	0.00	0.00	0.00
Southern Tablelands DSF	0.40	0.49	0.03	0.74	0.23	0.40	0.63	0.31	0.14	0.34	0.43	0.43	0.29	0.26	0.17
Southern Escarpment WSF	0.22	0.34	0.06	0.22	0.97	0.44	0.34	0.13	0.00	0.09	0.25	0.06	0.09	0.06	0.50
Montane WSF	0.34	0.37	0.17	0.31	0.54	0.80	0.43	0.23	0.06	0.20	0.29	0.09	0.03	0.00	0.23
Southern Tableland WSF	0.46	0.71	0.05	0.39	0.49	0.66	0.68	0.66	0.17	0.24	0.44	0.20	0.32	0.20	0.34
Sub‐alpine woodlands	0.70	0.54	0.14	0.43	0.32	0.78	0.51	0.46	0.14	0.32	0.43	0.19	0.19	0.14	0.30
Tableland Clay GW	0.35	0.46	0.05	0.22	0.24	0.43	0.38	0.68	0.43	0.14	0.27	0.08	0.38	0.14	0.16
Southern Tablelands GW	0.51	0.53	0.02	0.44	0.23	0.33	0.51	0.72	0.56	0.21	0.33	0.14	0.42	0.28	0.16
Temperate Montane grasslands	0.33	0.48	0.00	0.19	0.04	0.22	0.26	0.59	0.78	0.11	0.19	0.04	0.33	0.15	0.11
Southern Montane Heaths	0.28	0.17	0.03	0.38	0.14	0.10	0.45	0.10	0.03	0.66	0.38	0.34	0.10	0.07	0.03
Sydney Montane Heaths	0.17	0.13	0.00	0.13	0.04	0.04	0.67	0.04	0.04	0.25	0.92	0.29	0.04	0.00	0.04
Sydney Montane DSF	0.17	0.17	0.00	0.23	0.10	0.07	1.00	0.07	0.00	0.27	0.60	0.60	0.10	0.03	0.13
South East DSF	0.24	0.30	0.02	0.35	0.37	0.13	0.72	0.09	0.02	0.26	0.50	0.74	0.24	0.07	0.24
Montane Bogs and Fens	0.23	0.72	0.09	0.11	0.04	0.30	0.13	0.32	0.04	0.06	0.43	0.02	0.04	0.04	0.04
Montane Lakes	0.09	0.36	0.00	0.05	0.00	0.05	0.00	0.77	0.05	0.00	0.00	0.00	0.00	0.00	0.00
Central Gorge DSF	0.20	0.14	0.00	0.32	0.18	0.05	0.41	0.09	0.16	0.05	0.18	0.41	0.89	0.11	0.34

*Note*: Dark gray indicates proportions > 0.7 and pale gray proportions > 0.5. Cells with the same shading in column one comprise members of the same formation.

Abbreviations: DSF, Dry Sclerophyll Forests; GW, Grassy Woodlands; WSF, Wet Sclerophyll Forests.

**TABLE 6:** Proportion of species characteristic of each structural/physiognomic class that are diagnostic of units of a 15‐cluster solution derived using k‐means) (DSF ‐ Dry Sclerophyll Forests; WSF ‐ Wet Sclerophyll Forests; GW ‐ Grassy Woodlands)

Cluster	14	13	15	7	4	5	11	1	2	8	10	6	3	9	12
Alpine Herbfields	0.88	0.62	0	0	0.12	0.04	0.04	0.12	0	0.04	0.31	0.04	0.04	0	0.04
Alpine Bogs and Fens	0.43	0.43	0	0.04	0.21	0.11	0.07	0	0	0.04	0.18	0	0.07	0.04	0
Alpine Heaths	0.3	0.89	0	0.11	0.44	0.07	0	0.04	0	0	0.11	0	0	0	0
Alpine Fjaeldmarks	0.63	0.88	0	0	0	0	0.06	0.06	0	0	0.06	0	0	0	0
Southern Tablelands DSF	0	0	0.74	0.26	0.4	0.31	0.09	0	0.09	0.26	0.03	0	0.57	0.4	0.2
Southern Escarpment WSF	0	0	0.03	0.97	0.41	0.19	0	0	0.09	0.09	0.03	0	0	0	0
Montane WSF	0	0.09	0.09	0.57	0.8	0.23	0.06	0.03	0.14	0.06	0.14	0	0	0.06	0
Southern Tableland WSF	0	0	0.17	0.56	0.59	0.68	0.12	0	0.12	0.29	0.15	0.05	0.24	0.07	0.15
Sub‐alpine woodlands	0	0.16	0.27	0.38	0.84	0.49	0.11	0	0.08	0.16	0.14	0.03	0.19	0.11	0.08
Tableland Clay GW	0.03	0.05	0.08	0.27	0.41	0.62	0.46	0.03	0.03	0.32	0.16	0.08	0.3	0.03	0.14
Southern Tablelands GW	0	0	0.23	0.23	0.3	0.77	0.53	0	0.05	0.33	0.05	0	0.53	0.09	0.23
Temperate Montane grasslands	0.04	0.04	0.04	0.04	0.07	0.52	0.85	0	0	0.22	0.22	0.19	0.22	0	0.15
Southern Montane Heaths	0	0	0.52	0.07	0.14	0.1	0.03	0.34	0.24	0.07	0.07	0	0.21	0.28	0.07
Sydney Montane Heaths	0	0	0.13	0.04	0.04	0.04	0	0.88	0.67	0.04	0	0	0.04	0.25	0
Sydney Montane DSF	0	0	0.2	0.1	0.07	0.07	0	0.37	0.8	0.07	0	0	0.1	0.43	0.03
South East DSF	0	0.02	0.28	0.33	0.15	0.11	0	0.22	0.65	0.24	0.02	0	0.09	0.54	0.04
Central Gorge DSF	0	0	0.09	0.16	0.02	0.09	0.09	0	0.18	0.86	0	0	0.23	0.32	0.11
Montane Bogs and Fens	0.4	0.11	0.04	0.04	0.23	0.21	0.06	0.32	0.09	0.02	0.81	0.15	0.04	0	0.04
Montane Lakes	0.23	0.05	0	0	0.05	0.23	0.09	0	0	0	0.73	0.73	0.05	0	0

*Note*: Dark gray indicates proportions > 0.7 and pale gray proportions > 0.5. Cells with the same shading in column one comprise members of the same formation.

Abbreviations: DSF, Dry Sclerophyll Forests; GW, Grassy Woodlands; WSF, Wet Sclerophyll Forests.

## 4 DISCUSSION

### 4.1 Performance of alternative clustering methods applied to irregular data structure

Overall, the results of our analyses support both of our hypotheses; graph‐theoretic clustering produced less misplacement than central‐tendency clustering, particularly for broad groupings. Several caveats apply to this conclusion: (i) the utility of the different clustering methods cannot be encapsulated solely in terms of cluster homogeneity and rates of misplacement, (ii) internal evaluators can be misleading in terms of cluster quality, and (iii) the superior performance of Chameleon in elucidating upper‐hierarchical clusters is entirely dependent on selecting appropriate parameters from an infinite range of combinations. The clearest support for our hypotheses was evident in the comparison between solutions derived using Chameleon clusters with those derived by k‐means over the range from 15 to 250 clusters. Chameleon's best 15 and 30 cluster solutions exhibited significantly lower rates of misplacement than those of k‐means at the cost of an increase in heterogeneity (Figure 6), while at progressively higher numbers of clusters (60–250 clusters) there was a convergence in the respective metrics. We speculate that increasing rates of misplacement with finer sub‐division into clusters indicates the partitioning of a continuum. That is, when the data are partitioned into many, continuously intergrading communities, the proportion of their (ever decreasing) member sets, which most closely resemble samples in adjacent clusters increases. If there was indeed variability in the structure of our broad vegetation groups, then the algorithms performed as hypothesized. We conclude there was a clear advantage in using Chameleon over k‐means to elucidate our upper‐hierarchical clusters (and relatively little cost) but no apparent advantage in the derivation of larger numbers of clusters in terms of cluster metrics. However, since Chameleon solutions of progressively larger numbers of clusters can be produced by continually partitioning the sparse graph, the algorithm offers a straightforward method of integrating plot‐based classifications at multiple hierarchical scales.

Accounting for the performance of agglomerative and divisive clustering algorithms is more complicated. First, on the basis of cluster homogeneity and rates of misplacement, our agglomerative algorithm performed better than either Chameleon or k‐means, scoring higher on both metrics across the spectrum of solutions from few to many clusters, while our divisive algorithm scored worse (Figure 6). Both, however, produced 15‐cluster solutions of much greater unevenness in membership numbers than k‐means or Chameleon (Figure 7), which, if evidence of chaining (*sensu* Peet & Roberts, 2013), could suggest that both solutions were less informative in relation to the nature of upper‐hierarchical groupings. Conversely, our three traditional algorithms scored equally highly in terms of the number of diagnostic species and clearly higher than the best Chameleon solutions, suggesting that unevenness in cluster membership numbers could, in fact, be symptomatic of biases in the distribution of samples among “natural” clusters, and that the three traditional algorithms performed better in detecting these uneven clusters (as evidenced by higher numbers of diagnostic species).

Comparisons with a reference classification suggest unevenness in the cluster size is more likely to be indicative of chaining because the largest clusters were made up of samples representing multiple classes (as indicated by the range in diagnostic species), some of which are relatively distantly related. This phenomenon was most strongly evident in the agglomerative and divisive solutions (Tables 3, 4, 5). This reflects a well‐known weakness of agglomerative or divisive methods, which incorporate merge or split decisions based on the aggregate properties of clusters. Such methods require either unrealistic assumptions concerning the structure of the data and/or sequential merge/split decisions, which cannot be reversed and which are necessarily sensitive to the composition of the dataset (Han et al., 2012). While we did not evaluate the quality of solutions of greater than 15 classes, the agglomerative algorithm appeared to outperform all others in producing 250‐cluster solutions with low rates of misplacement and high homogeneity, although its subsequent, upper‐hierarchical groupings became progressively less meaningful because of poor merging decisions. We conclude that Chameleon and k‐means generated the most informative solutions of 15 clusters with the former perhaps better representing the natural structure of the data while the latter produced more homogeneous groupings.

### 4.2 Are “natural” clusters necessarily less homogeneous?

Although our approach trades‐off cluster homogeneity for improvements in (mis)placement of samples in the cluster, the degree of trade‐off is likely to depend on the structure of individual datasets. In our case study, the misplacement rate achieved by Chameleon was half that of k‐means at the cost of a 10% reduction in cluster homogeneity. If the clusters Chameleon retrieved in our dataset are indeed irregular shapes, then our results suggest they are unlikely to be highly elongated, and variability in our data structure tends toward uneven density rather than irregular shape.

The question of whether “natural” clusters necessarily have fewer diagnostic species is more difficult to resolve based on our analyses. *A priori*, we expect that more heterogenous clusters would mean fewer diagnostic species, the pattern reflected in our results. However, Schmidtlein et al. (2010) demonstrated that Isopam, an algorithm that adapts to irregular cluster shapes, consistently out‐performed other algorithms in terms of the number of indicator species (*sensu* Dufrêne & Legendre, 1997) and was also highly ranked in terms of the number of species with standardized phi > 0.35 (Tichý & Chytrý, 2006). Higher numbers of diagnostic species could reflect the sampling of a wider species pool, since samples sharing no species can occupy the same cluster if comprise an interconnected neighborhood (Schmidtlein et al., 2010). However, it is possible that higher numbers of diagnostic species are an artifact of Isopam's partitioning of the ordination space by medoids, notwithstanding the fact the ordination axes are adjusted in this method to accommodate nonlinearities. Further research is required into metrics to give insights into how well cluster solutions model the structure of vegetation data (e.g., within‐cluster interconnectedness, misplacement rates) to better understand the potential trade‐offs involved in maximizing homogeneity or indicator values.

### 4.3 Are natural clusters likely to be more stable/robust to new data?

Clustering solutions are notoriously sensitive to classification protocols, and it has generally proven difficult to retrieve prior classes via analysis of combined data (Tichý et al., 2014). Wiser and de Cáceres (2013) and Tichý et al. (2014) characterized this problem in terms of the need to preserve units of one or more Consistent Classification Sections (CCS, *sensu* De Cáceres et al., 2015) while allowing for previously unrecognized units to be identified following the acquisition of new plot data. To achieve this, they proposed alternative forms of semi‐supervised clustering as promising approaches that allow for units to be “fixed” by specifying their plot membership *a priori* while allowing unattributed plots to form new clusters. The question of when units should be “fixed” must still be addressed. Some understanding of the underlying data structure is likely to answer this question if the problem arises either from idiosyncratic clustering of irregular data or because of biases in the distribution of samples in compositional space.

In theory, algorithms sensitive to data structure may reduce the extent of this problem, at least at some level of data partitioning. Tozer et al. (2022) found that Chameleon's novel approach to modeling intersample relationships greatly facilitated the revision of an earlier broad classification of forested wetlands based on substantially fewer plot samples (Keith & Scott, 2005). Unlike many traditional methods, which incorporate merge or split decisions based on the aggregate properties of clusters, Chameleon operates on interconnected neighborhood sets. In Tozer et al.'s (2022) case, these were structured on the same similarity metric used in the original analysis. They considered these features pivotal because the algorithm could potentially minimize the impact of adding new data by retaining connections between samples from the original set (Tozer et al., 2022). Tozer et al. (2022) reasoned that since Chameleon dissolves connections between relatively weakly‐connected samples in the partitioning phases, strong pairwise relationships between samples underpinning clusters in the original analysis could be preserved (and reflected more faithfully) in their new Chameleon‐derived clusters.

We note that there is some uncertainty in relation to how the algorithm can be best implemented. We employed the Cluto clustering package (Karypis, 2003) distributed by Chameleon's authors; however, we noted some inconsistencies in relation to the parameters offered compared with the description of the algorithm (Karypis et al., 1999). Furthermore, Barton et al. (2019) have suggested that Cluto's implementation does not entirely embody the Chameleon concept. Barton et al. (2019) reproduced the results of Karypis et al. (1999) and developed an alternative implementation, which demonstrates improved performance, although it relies on a different partitioning algorithm because the original is proprietary protected.

## 5 CONCLUSION

Scale‐dependent irregularities in vegetation data can affect the utility and stability of clustering solutions underlying vegetation classification schemes. The existence of clusters of irregular shape and density implies that novel metrics are required in their evaluation because such clusters may not score well on traditional metrics that assume a spheroidal model (Aho et al., 2008). Evaluating the utility of such cluster solutions requires metrics that assess interconnectivity rather than central tendency.

Although our results support the theoretical notion that graph‐theoretic algorithms such as Chameleon are better suited to the task of elucidating vegetation classes, the trade‐offs in its solutions, and the ways in which these improve upon those retrieved by traditional clustering approaches require further quantification. We suggest this is a worthwhile endeavor because Chameleon offers a conceptually simple model, can process very large datasets quickly, and potentially presents a solution to the problem of integrating plot‐based classifications across hierarchical levels.

## AUTHOR CONTRIBUTIONS

**Mark Tozer:** Conceptualization (equal); data curation (equal); formal analysis (equal); investigation (equal); methodology (equal). **David A. Keith:** Conceptualization (equal); project administration (equal); supervision (equal).

## ACKNOWLEDGEMENT

This research was supported by an Australian Government Research Training Program scholarship. We thank Dr Kate Wilson, former Executive Director of Science, NSW Department of Environment and Principal Research Scientist Dr Tony Auld for their support and constructive discussions at the inception of this project. We acknowledge the considerable contribution made by Ken Turner to data curation and thank Greg Steenbeeke for digitising the NSW state vegetation classification species lists. This work has been greatly improved by the inciteful and constructive suggestions we received from our reviewers and editors.

## Data Availability

CLUTO software modules are available for download from Karypis Lab website (http://glaros.dtc.umn.edu/gkhome/cluto/cluto/download ). A more recent implementation of Chameleon algorithm in JAVA is included as a module in the clustering platform Clueminer (https://github.com/deric/clueminer ). Plot data used in our analyses are available at: https://www.environment.nsw.gov.au/research/Vegetationinformationsystem.htm (NSW DPIE 2020, accessed 2nd August 2016). All analyses were performed on a matrix of similarity (1‐Bray‐Curtis dissimilarity) between the objects to be clustered. Data were imported in a plain text file with *n* + 1 lines, the first line containing the number of rows, and the remaining *n* lines containing similarity values for each row (Karypis, 2003).
